# Understanding Wisdom: An Investigation of Gender Difference in Old Age

**DOI:** 10.1093/geroni/igaa057.1524

**Published:** 2020-12-16

**Authors:** Kathryn Hartikka

**Affiliations:** University of Florida, Gainesville, Florida, United States

## Abstract

Understanding wisdom contributes to a larger dialogue on how best to serve older adults as they go through the aging process. Namely, by identifying gender differences in wisdom conceptualization, research can further gather information regarding the complex, implicit definitions. To investigate how older men and women differ in conceptualizing wisdom, semi-structured qualitative interviews with seven men and eight women (age range: 64-86 years) who scored above average on the Three-Dimensional Wisdom Scale (3D-WS), the Adult Self-Transcendence Scale (ASTI), and the Foundational Values Scale (FVS) were compared. In-depth analysis of semi-structured interviews uncovered spiritual and non-spiritual experiential-based knowledge, “good” or spiritual-based decision-making, and a selfless care for others as common themes among women. Men invoked similar themes, such as spiritual-based decision-making and experiential-based knowledge, yet also differed in conceptualizing wisdom by emphasizing themes such as growth through hard times and overcoming obstacles. Men also considered wisdom to be related to an open-mindedness about life and rarely noting selfless care for others as a characteristic of wisdom in contrast to female respondents. The findings confirm earlier quantitative research results on implicit wisdom theories that men are more likely than women to have a cognitive understanding of wisdom, whereas women are more likely than men to characterize wisdom as an integrative construct.

